# Radiographic Evaluation of Keel Bone Damage in Laying Hens—Morphologic and Temporal Observations in a Longitudinal Study

**DOI:** 10.3389/fvets.2020.00129

**Published:** 2020-03-12

**Authors:** Sarah Baur, Christina Rufener, Michael J. Toscano, Urs Geissbühler

**Affiliations:** ^1^Clinical Radiology, Department of Clinical Veterinary Medicine, Vetsuisse Faculty, University of Bern, Bern, Switzerland; ^2^Center for Proper Housing: Poultry and Rabbits, Animal Welfare Division, Veterinary Public Health Institute, Vetsuisse Faculty, University of Bern, Bern, Switzerland

**Keywords:** fracture, aviary system, imaging, age, x-ray

## Abstract

The keel bone of commercially kept laying hens is known to be frequently affected by morphologic changes such as fractures and deformations with important implications for animal welfare. To detect morphologic changes, various methods such as palpation, computed tomography, and ultrasound are available, though radiography allows for the greatest level of detail in combination with the most ease of use. To explore the benefits of radiography in providing objective data on keel fractures from the age of 22–61 weeks within a single laying period, the keel bones of 75 Lohmann Brown and 75 Lohmann Selected Leghorns were radiographed every 3 to 5 weeks. Type, location, angulation, dislocation, callus formation, and healing process were assessed descriptively for each lesion. Ninety-nine percent of the animals showed at least one keel bone lesion during the study and 97% of the animals had at least one keel bone fracture. In 77% of the cases, the caudal third of the keel bone was affected. The fracture types were transverse and oblique (88%), comminuted, and butterfly. Further lesions were sclerosis, new bone formation and angulation. For each keel bone, an average of three fractures (3.09 ± 1.80) was detected at the end of the study. The described radiographic protocol for keel bone lesions was suitable for longitudinal, on-site examinations in conscious laying hens. Our results also indicate that keel bone fractures are more frequent than reported in earlier studies. The described radiographic examination protocol can be used to perform comparative studies of palpatory findings, or to assess the clinical significance of different fracture types which require a high level of detail.

## Introduction

Housing of laying hens for egg production is known to be associated with skeletal problems such as fractures and deformities of the keel bone (1). The causes of such fractures have not been definitively identified but are suspected to be a multifactorial problem including: genetic regulation of bone health and high egg laying performance (2, 3), bone calcium depletion, and collisions within the housing systems (4–6).

The most common method for evaluating whether laying hens have keel bone fractures (KBF) is palpation, a relatively simple and low-cost method that allows for longitudinal observations. Despite these benefits, palpation requires assessors to undergo training and evaluation to ensure reliable and accurate results (4, 7). Even with superior training, it is likely that a large percentage of fractures will be missed due to a variety of reasons including: fissures, inability to detect fractures on the dorsal aspect of the keel, or damage hidden by the large breast muscle group.

Due to these concerns, alternative techniques should be evaluated that would allow for reliable, longitudinal assessment of KBF. Radiography, a well-established method for fracture detection (8), has been used to detect KBF in several, non-commercial (9–11) as well as quasi-commercial (12) settings.

An evaluation protocol was developed for scoring gross severity of fractures (available at: http://www.keelbonedamage.eu/activities/practical-information-for-stakeholders/online-tool-for-evaluating-fractures-from-radiographic-images/>) that was determined to be reliable in terms of intra- and inter-observer reliability (12). The protocol was successfully used to grade fractures in relation to hen productivity (12) and mobility (13). Although Rufener et al. (12) included presence of a fracture gap to indicate healing, the protocol was fairly narrow in scope. For instance, the protocol did not classify the location, type, or size of the fracture. Given the variation of these fracture features in commercial laying hens, it is likely that damage will have variable effects on hen welfare and productivity where detailed information about fracture characteristics might aid disentangling aspects of clinical significance. For instance, it is unknown if small fractures at the caudal tip are associated with pain differently than fractures similar in size but located on the cranial aspect of the keel. In the same vein, different types of fractures may be associated with different causes, e.g., external forces resulting in traumatic injuries vs. pathologic causes due to reduced bone strength (1). Without an objective classification of KBF, efforts at linking the causes and effects of different types of fractures are severely hindered.

The aim of this study was to describe a radiographically based, objective, fracture-specific characterization of KBF. Our methodology specifically is intended for longitudinal observations and considers changes in individual fractures over time, specifically a 40-week interval within a single laying period.

## Materials and Methods

The study was performed in a barn managed by the Aviforum (www.aviforum.ch), a contract research facility that owned the animals and was the sole provider of animal care. All animals were sourced from a commercial hatchery and then slaughtered at an abattoir per common industry practice at the conclusion of the laying period. Under a long-standing agreement between the Aviforum and the Center for Proper Housing of Poultry and Rabbits, 10 individual compartments were used for the study. In each of the 10 compartments, 15 animals from one genetic line [i.e., 5 compartments with 15 Lohmann Brown (LB) animals and 5 compartments with 15 Lohmann Selected Leghorns (LSL) animals] were maintained alongside 210 laying hens of the other hybrid. All the animals were of the same age. Full detail of the barn configuration and management protocols, including rearing, are provided by Rufener et al. (14). A total number of 75 LB and 75 LSL, each of them individually marked with a number at the phalanges, were included in the longitudinal study. All the hens were kept under the same conditions in a Bolegg Terrace aviary system.

The animals were examined over a period of 10 months during which each animal was assessed 11 times (22, 25, 28, 33, 37, 40, 45, 49, 54, 57, and 61 weeks of age). For better understanding, the term “observation Phase (P)” from 1 to 11 is used hereafter. On the examination day, focus animals were caught, placed in transport boxes, and moved to the radiography site within the barn (i.e., within the hygiene barrier), a distance varying between 10 and 30 m depending on the location of the pen. The hens remained conscious and were not anesthetized for the entire procedure.

**Figure 1:**
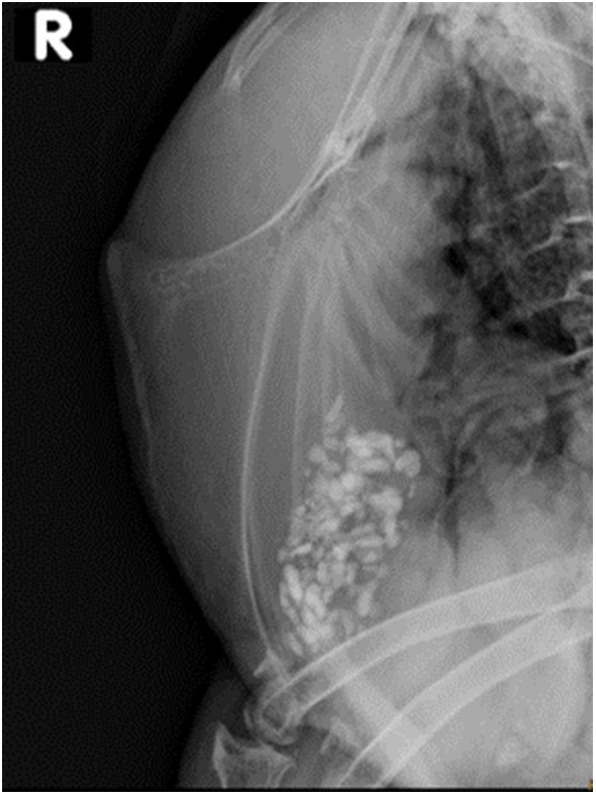
Laterolateral view of the keel bone of the free-range laying 10 years old Appenzeller Spitzhaubenhuhn “Esmeralda” without visible fractures or lesions. Limited interpretation of the caudal keel bone tip due to superimposition of one stifle.

Based on pilot trials (10), the following slightly adapted radiographic protocol was used. A single laterolateral radiograph of the keel bone (Figure 1) was performed with a portable X-ray machine consisting of a focal spot of 1.2 × 1.2 mm (Gierth X-Ray international GmbH), and 46 kV, 2.4 mAs and a focus-film-distance of 80 cm were set. The imaging system consisted of a flat panel detector (CXDI-50G, Canon). The conscious hens were positioned as described by Sirovnic et al. (10). As the size of each hen within a hybrid was consistent, the exposure field had to be readjusted only between the different hybrids. After exposure, the animal was immediately returned back to the crate where it remained until the procedure was completed for all animals, whereupon hens were returned to their home pen. The entire process, including collection of hens, imaging, and returning the animals, took approximately 120 min for 75 hens. A radiation protection permission was granted by the Swiss authority for this study (approval number BE-03222.41.013). Approval for use of experimental animals was obtained from the Veterinary Office of the Canton of Bern in Switzerland (approval number BE31/15). The experiment complied with Swiss regulations regarding the treatment of experimental animals.

The images were imported into the Picture Archive and Communication System (IMPAX EE, Agfa Healthcare) of the Vetsuisse Faculty of Berne and evaluated on a radiographic workstation with a certified medical screen (EIZO) with a DICOM radiology evaluation software (IMPAX EE Client, Agfa Healthcare). All evaluations were conducted by a single person (SB) with guidance as needed (UG). The morphology, location, time of first appearance and the change over time of each lesion were reported. Additionally, the soft tissues around the keel bone were evaluated.

**Figure 2:**
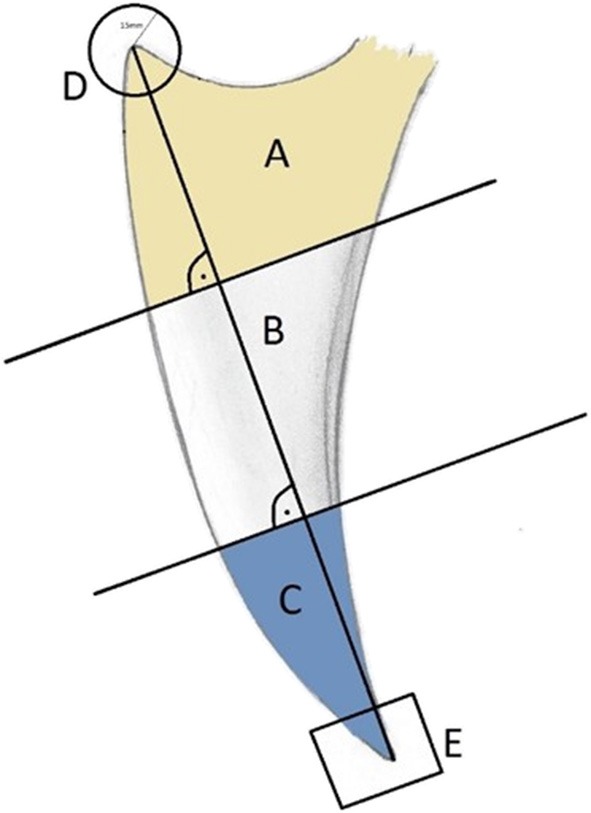
Laterolateral view of a schematic representation of a keel bone. The keel bone is divided in three parts, each of them measures one third of the base to apex line: Cranial third **(A)**, middle **(B)**, and caudal third **(C)**. The apices are labeled **(D)** (cranial; *r* = 15 mm) and **(E)** (caudal 1/5 of **C**).

The keel bone was initially divided into thirds: A (cranial), B (middle), and C (caudal). In addition, the cranial aspect was denoted by “D” (circle with a radius of 15 mm and a center at the cranioventral tip of the keel bone) and the caudal aspect denoted by “E” (caudal fifth of the caudal third C; Figure 2). Fractures and other lesions in “E” were attributed to “C” if not specified. Fractures and other lesions in “D” were attributed to “A” if not specified. Furthermore, it was noted if a lesion involved the dorsal, ventral, cranial or caudal bone surface.

**Figure 3:**
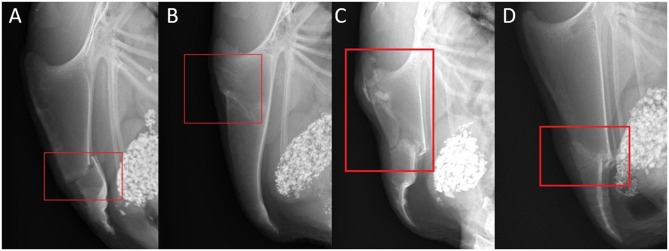
Examples of fracture and lesion types and localizations. **(A)** Laterolateral view of a complete transverse keel bone fracture in the caudal third (localization C) with caudodorsal dislocation and angulation. **(B)** Laterolateral view of two incomplete oblique keel bone fractures in the cranial third (localization A) with a ventral superficial step formation and slight ventral angulation at the caudal fracture. **(C)** Laterolateral view of a comminuted keel bone fracture in the cranial and middle third (localization AB) with a ventrocranial dislocation and angulation of the caudal main fragment. **(D)** Laterolateral view of a butterfly keel bone fracture in the middle and the caudal third (localization BC) with ventral dislocation and angulation of the butterfly and caudal main fragment.

A bone lesion could be either fracture or not fracture-related. A lesion was defined as a fracture if a step at the bone surface or a fracture gap was present. The fractures were divided into categories: transverse, oblique, butterfly, and comminuted. Non-fracture related lesions were sclerosis, new bone formation without a fracture gap, and angulation (Figure 3). A transverse fracture was noted, if the fracture line did not deviate more than 10 degrees from the perpendicular line to the base to apex line of the keel bone. If the angle measured 11 or more degrees, an oblique fracture was noted. A butterfly fracture was noted if three main fragments were present and the middle main fragment was roughly triangular. All other fractures with more than two fragments were reported as comminuted fractures. Sclerosis was reported if a bone opacification was detected. A new bone formation was noted in case of superficial new bone formation without the presence of a fracture gap. An angulation was noted in case of a change of the axis within the keel bone.

A healing of a fracture was noted when the fracture gap was no longer visible in subsequent radiographs. It could be accompanied by callus formation. A fracture was also considered to be healed if no gap or superimposition was visible at the time of the first onset, even if a step at the bone surface was present. In addition, the length of each keel bone from the caudal to the cranial tip was measured.

The present study was of an explanatory nature, with the aim to characterize and describe KBF and their change over time, as well as develop a standardized methodology to accomplish said aim. Given these objectives, our methods employed primarily descriptive statistics to describe the temporal occurrence of lesions and fractures, their characteristics (e.g., localization, type, callus formation, soft tissue swelling), or the proportion of hens being affected by a certain type of lesion/fracture. In addition, we grouped collected data at the hen-level to evaluate whether fracture development differed over time between LB and LSL hens using a linear mixed effect model [package “lme4” (15)] in R 3.4.2 (16). The outcome variable was the total number of fractures per hen and phase. Age (continuous), hybrid (factor with 2 levels: LB, LSL) and their interaction were included as fixed effects, and hen nested in pen was used as a random effect. Model assumptions (normality of errors and homoscedasticity) were checked through graphical analysis of residuals. The final model was obtained by a stepwise backwards reduction using parametric bootstrap tests [package “pbkrtest” (17)] for model comparison and a *p* > 0.05 as the criterion for exclusion. The package “effects” (18) was used to calculate and display model estimates.

## Results

### Radiographic Procedure

The mortality rate [percentage of dead hens/hens housed/4 weeks (19)] was 0.38% for focus birds (*n* = 8) and 0.87% for non-focus birds prior to the last radiographic timepoint at 61 weeks of age. No animals died during the procedure including: catching and transporting the birds from the housing system to the examination area, during suspension, or while returning them to the aviary system.

Following catching of the hens and placement in lairage crates, the procedure for each hen was approximately 60 s in duration beginning with removal from the crate, suspension, radiographic exposure and image collection, and finally returning the hen to the crate. During the first session, five out of 75 X-rays had to be repeated due to poor image quality (i.e., motion unsharpness). For the remaining sessions, approximately one radiograph per session had to be repeated due to poor image quality.

### Lesion Incidence

A total of 544 lesions including 422 fractures occurring on all 150 keel bones were identified; 99% of the hens had at least one keel bone lesion during the study period. Seventy-eight percent of the lesions were attributed to a fracture and 97% of the X-rayed animals had at least one KBF throughout the course of the study. Thirty percent of the hens had three KBF (mean ± SD for the whole database: 3.09 ± 1.80) at the end of the study with a maximum of 15 lesions (Table 1) and 11 fractures (Table 2) per keel bone observed. Keel bone lesions, which were not related to a fracture, hereafter referred to as non-fracture lesions, occurred in 22% of described lesions in this study. Of these, 39% were sclerotic areas, 39% angulations, 15% new bone formations, 6% indentations, and 1% other deformations. The number of new lesions per localization and hybrid is shown in Table 3.

**Table 1:** Mean, standard deviation (STD), minimum, and maximum value of total number of lesions per hybrid and time point.

	**LB**				**LSL**			
**Phase**	**Mean**	**STD**	**Min**	**Max**	**Mean**	**STD**	**Min**	**Max**
P1	0.1	0.3	0	2	0.1	0.3	0	1
P2	0.3	0.5	0	2	0.4	0.7	0	2
P3	0.5	0.8	0	3	0.9	0.9	0	3
P4	1.1	1.1	0	5	1.4	1.1	0	4
P5	1.8	1.3	0	5	2.0	1.2	0	4
P6	2.4	1.6	0	7	2.3	1.2	0	5
P7	2.8	1.8	0	9	2.5	1.2	0	6
P8	3.3	2.1	0	11	2.7	1.2	1	6
P9	3.6	2.2	0	13	2.9	1.2	1	6
P10	3.8	2.2	0	13	3.1	1.2	1	6
P11	4.0	2.4	0	15	3.2	1.2	1	6

**Table 2:** Mean, standard deviation (STD), minimum, and maximum value of total number of fractures per hybrid and time point.

	**LB**				**LSL**			
**Phase**	**Mean**	**STD**	**Min**	**Max**	**Mean**	**STD**	**Min**	**Max**
P1	0.1	0.2	0	1	0.0	0.2	0	1
P2	0.2	0.4	0	2	0.3	0.6	0	3
P3	0.4	0.7	0	3	0.7	0.9	0	4
P4	0.9	1.0	0	5	1.1	1.0	0	4
P5	1.6	1.3	0	5	1.5	1.1	0	4
P6	2.1	1.5	0	6	1.7	1.1	0	4
P7	2.5	1.7	0	7	1.9	1.2	0	5
P8	2.9	1.9	0	8	2.0	1.1	0	5
P9	3.2	2.0	0	9	2.2	1.2	0	5
P10	3.4	2.0	0	9	2.3	1.2	0	5
P11	3.6	2.1	0	11	2.5	1.2	0	5

**Table 3:** Absolute number of new lesions per localization for each phase and hybrid.

			**Number of new lesions per localization**										
**Hybrid**	**Phase**	**# hens**	**A**	**A-B**	**A-B-C**	**A-D**	**B**	**B-C**	**C**	**C-E**	**D**	**E**	**Total**
LB	P1	75	2	1		1			1		1	1	7
	P2	75	2						8		1		11
	P3	75	1				2		16		1	1	21
	P4	74	7				3		35		1	6	52
	P5	74	2	3	1				32		1	4	43
	P6	74	1						19			14	34
	P7	74	4	2	1		3		23	1	1	9	44
	P8	74	3	2			5		14		1	7	32
	P9	73	2	2			1	1	11		3	6	26
	P10	72		2			2		8		2	3	17
	P11	72				1	1		10		2	3	17
LSL	P1	75		3					4	1		1	9
	P2	75	1	1					16			3	21
	P3	75	2	1					19	1	2	12	37
	P4	75	3	2					25	1	1	18	50
	P5	75	6	2					15	1		5	29
	P6	75	2	3			2	1	12	1		4	25
	P7	74	2	1			1	1	5			4	14
	P8	74	5	3			1	1	5			3	18
	P9	73	4	2			3		4	1		3	17
	P10	72	2	1					4		1	1	9
	P11	70	7	2					1			1	11
Total			58	33	2	2	24	4	287	7	18	109	544

*Total number of hens and total number of new lesions are given for reference. One individual hen could be affected by multiple new lesions in the same or multiple localizations*.

### Fracture Types, Temporal Detection, and Development

The most frequent fracture types were transverse (45% of all fractures) and oblique (43% of all fractures). Comminuted fractures were present in 11% and butterfly fractures in 1% of all identified fractures. A considerable amount of fractures showed dislocation (34.8%), angulation (52.8%), or both (23.7%). Angulation occurred roughly equally in the dorsal (25%) and ventral (26%) directions. In about 4% of fractures, the direction of angulation changed between subsequent observation periods.

**Figure 4:**
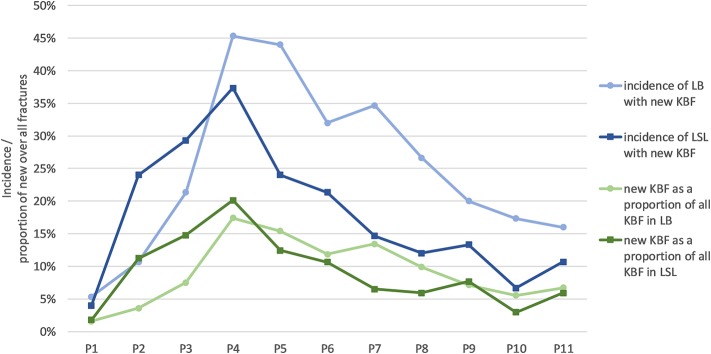
Incidence of hens with new fractures per phase (blue lines) and new fractures in proportion to all fractures per phase (green lines) for LB (Lohmann Brown) and LSL (Lohmann Selected Leghorn) hens.

**Figure 5:**
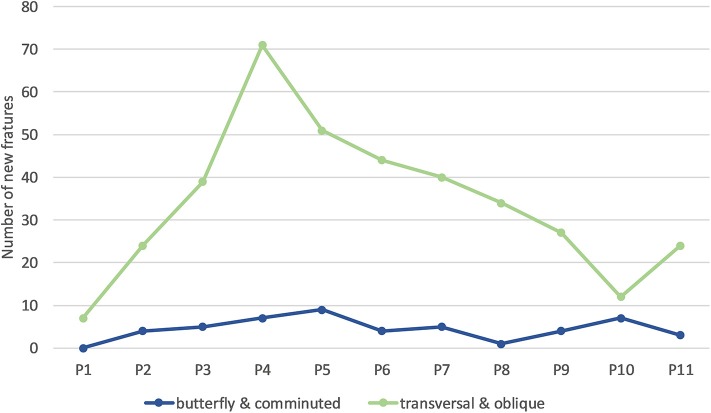
Number of new keel bone fractures of different types occurring per phase across all hens. One individual hen could be affected by multiple new fractures of the same or different types.

**Figure 6:**
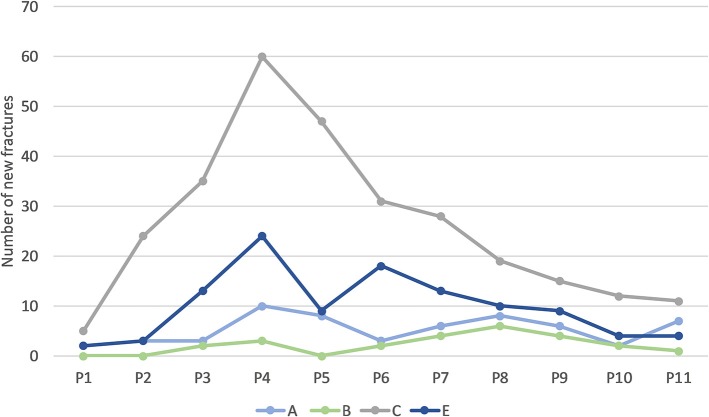
Number of new lesions (including fractures and non-fracture lesions) in different bone areas occurring per phase across all hens. One individual hen could be affected by multiple new fractures in the same or in multiple areas.

Most fractures occurred between 28 and 37 weeks of age (P3–P5; Table 4), with a peak during P4 when 45% of LB and 37% of LSL hens acquired a new fracture. During P4, as a percentage of all fractures within a genetic line, 17 and 20% were observed in LB and LSL, respectively (Figure 4). There was a peak in new transverse and oblique fractures as well as new comminuted and butterfly fractures between week 33 and 37 (P4, P5). Whereas, the occurrence of new transverse and oblique fractures seemed to decrease rather linearly after P4, the occurrence of butterfly and comminuted fractures was more variable (Figure 5, Table 5). The areas A, C, and E were affected by new lesions (fracture and non-fracture related lesions) mainly between week 31 and 33 (P4) (A: 17% of all lesions in A, C: 21% of all lesions in C, E: 22% of all lesions in E), whereas the occurrence of fractures in other localizations did not seem to be related to specific observation phases (Figure 6, Table 4).

**Table 4:** Absolute number of new fractures per localization for each phase and hybrid.

			**Number of new fractures per localization**										
**Hybrid**	**Phase**	**# hens**	**A**	**A-B**	**A-B-C**	**A-D**	**B**	**B-C**	**C**	**C-E**	**D**	**E**	**Total**
LB	P1	75	1	1					1		1		4
	P2	75	2						6		1		9
	P3	75	1				1		16		1		19
	P4	74	4				3		31		1	5	44
	P5	74	2	3	1				29		1	3	39
	P6	74	1						18			10	29
	P7	74	3	1	1		1		21	1	1	5	34
	P8	74	2	1			2		13		1	7	26
	P9	73	2	1			1	1	9		1	3	18
	P10	72		1			2		8		1	2	14
	P11	72				1	1		10		2	3	17
LSL	P1	75							2			1	3
	P2	75		1					16			2	19
	P3	75	1	1					17	1		5	25
	P4	75	1						22		1	10	34
	P5	75	3	2					14	1		1	21
	P6	75	1	2			2	1	11	1			18
	P7	74	2				1	1	4			3	11
	P8	74	2	2			1		4			1	10
	P9	73	3	2			2		4	1		1	13
	P10	72	1						3		1		5
	P11	70	7	2					1				10
Total			39	20	2	1	17	3	260	5	13	62	422

*Total number of hens and total number of new fractures are given for reference. One individual hen could be affected by multiple new fractures in the same or multiple localizations*.

**Table 5:** Absolute number of new fractures per fracture type for each phase and hybrid.

			**Number of new fractures per fracture type**					
**Hybrid**	**Phase**	**# hens**	**Butterfly**	**Greenstick**	**Oblique**	**Comminuted**	**Transverse**	**Total**
LB	P1	75			2		2	4
	P2	75			1	3	5	9
	P3	75			7	4	8	19
	P4	74		2	13	5	24	44
	P5	74	2	1	7	4	25	39
	P6	74		3	14	2	10	29
	P7	74		2	15	3	14	34
	P8	74		2	12	1	11	26
	P9	73		2	8	2	6	18
	P10	72	1		8	4	1	14
	P11	72			10	2	5	17
LSL	P1	75					3	3
	P2	75			9	1	9	19
	P3	75		1	9	1	14	25
	P4	75			9	2	23	34
	P5	75			9	3	9	21
	P6	75		1	7	2	8	18
	P7	74	1	1	1	1	7	11
	P8	74			7		3	10
	P9	73		2	7	2	2	13
	P10	72			1	2	2	5
	P11	70			9	1		10
Total			4	17	165	45	191	422

*Total number of hens and total number of new fractures are given for reference. One individual hen could be affected by multiple new fractures in the same or multiple localizations*.

Fifty-four percent of all lesions, which were initially not described as a fracture, fractured in the following observation period, leading to a total of 461 fractures. Forty-six percent of the initially described scleroses fractured in a later observation period.

In 84% of fractures, complete healing was diagnosed. Of these, 20% were already entirely healed at the time of detection and 43 and 22% healed within one or two observation periods, respectively. Less than 11% of the complete healed fractures required between 12 and 32 weeks until the fracture was healed as determined by radiography. In 16% of fractures, no healing could be detected.

**Figure 7:**
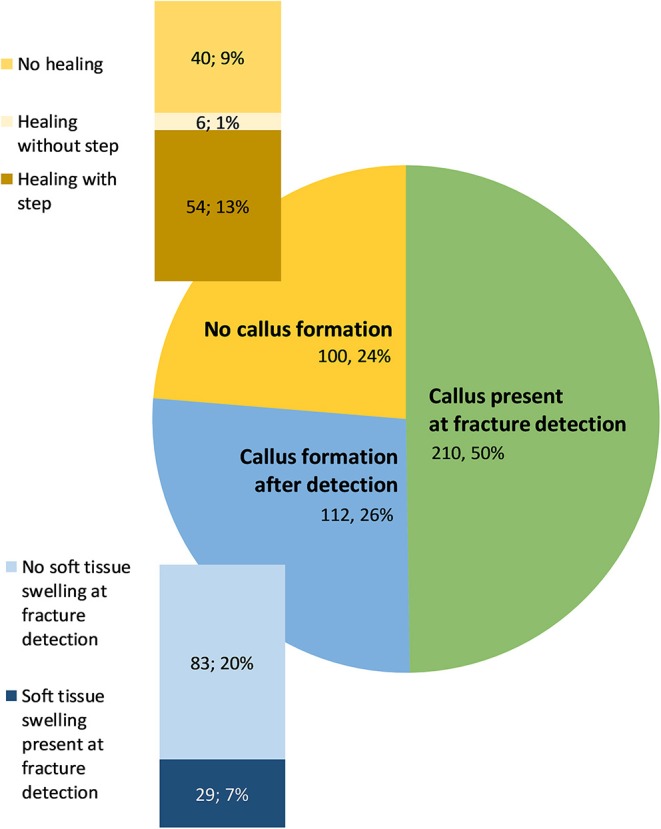
Absolute numbers and proportion of fractures with callus present at fracture detection, after fracture detection, or forming no callus. Within the fractures without callus formation, the absolute number and of proportion of fractures with different healing processes is shown. Within the fractures with callus formation after detection, the absolute number and proportion of fractures with and without soft tissue swelling is given.

In 76% of fractures, a callus was formed; in 50% a callus was already present at the time of fracture occurrence, whereas in 26% of new fractures the callus appeared at a later observation period (Figure 7). Of these, 26% (i.e., 7% of all fractures) showed a soft tissue swelling at the time of fracture detection. Of the 24% of the fractures without callus, the fracture gap completely disappeared over time and no step at the bone surface was visible in 13% of the cases, whereas the gap disappeared but a step at the bone surface was visible in a later observation time in 1% of all fractures. Nine percent manifested no fracture healing at all. The majority of the keel bones shortened during the observation period (65%). The length of the keel did not change in 9% and increased in 26%.

### Fracture Location

**Figure 8:**
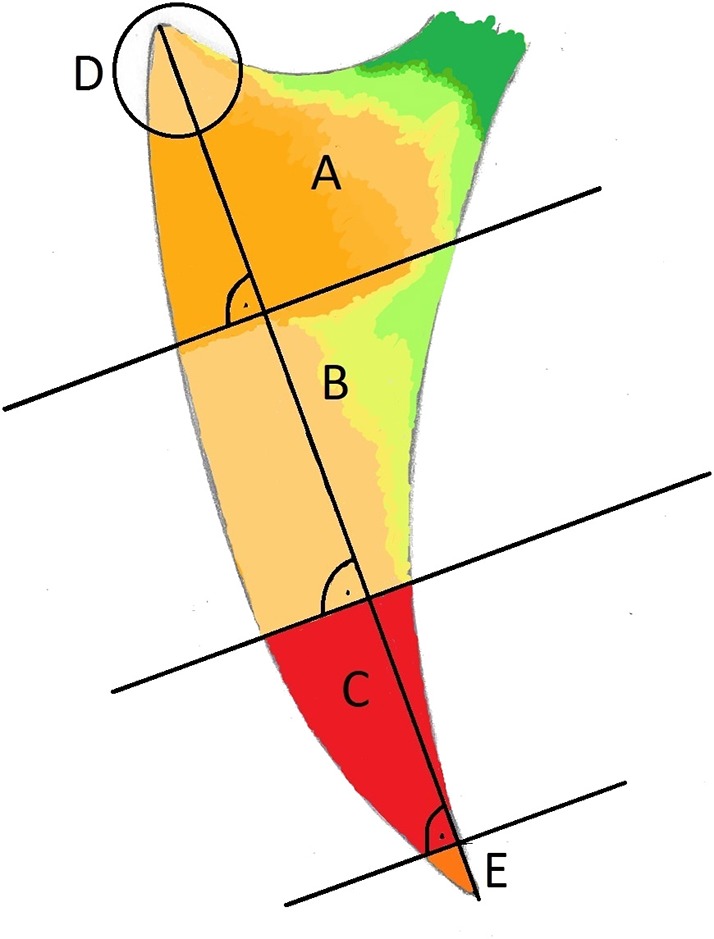
Subjective schematic visualization of the spatial frequency of new keel bone fractures: Low (green), moderate (yellow) and high (red) frequency. **(A)** Cranial third, **(B)** middle and **(C)** caudal third. The apices are labeled **D** (cranial; *r* = 15 mm) and E (caudal 1/5 of **C**).

From all fractures, 62% were localized in section C, 15% in E, followed by sections A, AB, B, D, BC, and CE (Figure 8, Table 4). From all non-fracture lesions, 39% were localized in section E, 22% in C, 16% in A, 11% in AB, followed by B, D, CE, BC and AD (Table 3). The most common lesion types depending on location were: sclerosis in 38% of non-fracture lesions in section A, angulation in 92% of non-fracture lesions in section E, new bone formation in 56% of non-fracture lesions in section C, and indentations in 86% of non-fracture lesions in section AB.

Sixty-eight percent of KBF extended the entire height of the keel, running from the ventral to the dorsal or from the ventral to the cranial bone surface. Ninety-seven percent of all fractures in section A, 95% of all fractures in B, 71% of all fractures in AB, and 60% of all fractures in CE did not extend the entire height. All fractures in sections AD and ABC, 67% of all fractures in BC, 90% of all fractures in E, 79% of all fractures in C and 77% of all fractures in D did run the entire height.

Transverse and oblique fractures were mostly localized in section C (60%). Comminuted and butterfly fractures were predominantly localized in section C (73%), but also occurred in ABC (4%).

### Genetic Lines

**Figure 9:**
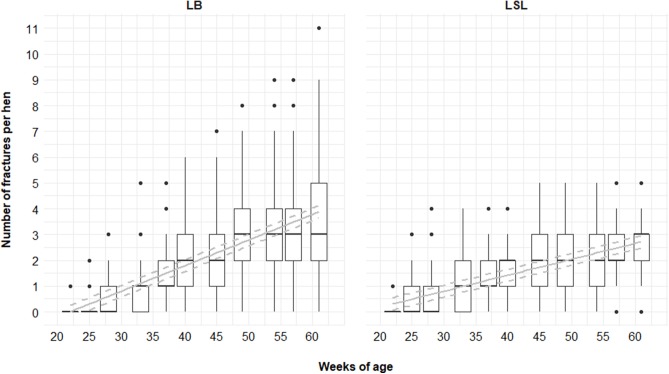
Total number of fractures per hen and phase for LB (Lohmann Brown) and LSL (Lohmann Selected Leghorn) hens. Boxplots show medians, interquartile and absolute ranges of raw data plus outliers. Solid lines represent estimated means, dashed lines indicate the 95% confidence interval.

In relation to genetic line, 95 and 99% of the LSL and LB animals, respectively, had at least one fracture. Sixty percent of all fractures were found in LB hens (and 40% in LSL). An average of 2.52 ± 1.20 (mean ± SD) and 3.63 ± 2.14 of fractures per keel bone were found for LSL and LB hens, respectively (Table 2). A maximum of 5 and 11 fractures per keel bone for LSL and LB hens, respectively, were visible. The increase in the total number of fractures per hen was steeper for LB hens than for LSL hens (p _hybrid*age_ < 0.001; Figure 9). Accordingly, the average number of KBF per hen was higher in LSL hens until 33 weeks of age (P4), whereas LB hens had more fractures than LSL hens in P5–P11 (Table 2).

### Soft Tissue Swelling

In 25% of the fractures, a soft tissue swelling was detected at the first time being observed. In 69% of comminuted fractures, a concurrent soft tissue swelling was present. Butterfly fractures showed soft tissue swelling in 25% and transverse and oblique fractures in 21% of the cases at the first time being observed.

## Discussion

### Study Value

To the author's knowledge, the current manuscript is the first analysis of radiographed keel bone damage that includes detailed information regarding damage morphology and development along an entire production period of laying hens. The radiographic procedure was rapidly performed in the commercial environment and generated relatively high-quality images without accidental radiation exposure, thus we believe our protocol can effectively deliver accurate representations of the keel suitable for future research efforts. By combining objective assessment of individual facets of keel damage with the detail of repeated radiographic assessments over a 40 week period, our assessment protocol provided several findings that will improve understanding of KBF and its causes. We believe these benefits are afforded directly by the level of detail provided by radiography which would not be possible with other common methods of assessment, i.e., palpation or dissection.

### Fracture Incidence

For instance, a relatively surprising result that appears to conflict with earlier reports are the sheer number of hens that manifested fracture or damage of some type. In this study, 97.0% of the animals had at least one fracture and 99.3% had at least one lesion at the end of the study (61 weeks of age). Previous studies have suggested less frequent occurrence (5, 20–24) with typically 50–80% of surveyed hens manifesting keel fractures by the end of lay using palpation or dissection. As a potential explanation for the relatively higher frequency seen in the current study, our protocol allowed for recent, healed, and minor fractures to be diagnosed (7, 9) which could be missed with palpation and/or dissection. Alternatively, flock and facility differences could have played a role as our observations where conducted within a single barn, though previous efforts in the same barn with assessment by dissection also found a lower frequency of fracture (25). Nonetheless, our findings suggest the problem may be more severe than previously thought and highlight the need for reliable metrics when assessing KBF that can be compared across varying conditions and situations. Given that the radiographic procedure can be performed rapidly in both commercial and non-commercial environments to generate relatively high-quality images, we believe our protocol should be adopted for future efforts.

In our study, 76% of the fractures developed a callus which is one of the main features recognized during palpation. However, at the time of detection, only 49% of the fractures had callus formation and, in 24% of the fractures, no callus was visible of which 95% showed no soft tissue swelling. In the absence of palpable indicators of damage such as soft tissue swelling, crepitation, angulation/dislocation of fragments, and callus formation, diagnosing fractures by palpation is impossible, where especially acute fractures without soft tissue swelling and/or crepitation could be missed leading to a false negative result. Some of the non-fracture lesions were also associated with soft tissue swelling, which could be misinterpreted as a fracture by palpation, i.e., a false positive result. Clearly, radiography can be assumed to be far more sensitive to aspects of fracture than palpation providing substantial benefits to detection efforts. Radiography also proved to be effective in assessing damage that might not be possible by palpation because of location due to muscle mass especially in the cranial (A and B) and dorsal portions.

### Fracture Location and Type

In addition to specific features of KBF that our protocol could characterize, it also afforded the ability to distinguish the fracture types and location. Fractures on the keel bone were predominantly transverse and oblique and were localized in area C (caudal third), an area recognized as the most frequently affected (20, 23). Although this study was not intended to assess the causes of observed damage, characterizing KBF features in this manner could aid in this process and should be considered. For instance, a possible explanation could be that section C the keel bone has less stability due to the anatomically reduced diameter in contrast to areas A or B making the area more susceptible to fracture. However, this would not explain why the apices E and D were affected much less frequently than C. Another explanation to explain the differential frequencies of damage in these areas could be related to the muscling of the animals. On the keel bone, the flight muscles of the animals are attached laterally and large muscling in areas A and B might absorb external impact forces which occur in the case of a collision. As area C is less muscled (26), this section may lack the capacity to absorb external forces. Another explanation for the high rates of damage in section C may involve the presence of internal organs like the gizzard which lies directly dorsal to the keel and is relatively rigid with high resistance (27). As a force applied to the keel is absorbed by underlying compressive tissue such as air sacs, area C might be more susceptible to fracture during external impact due to the collision with the incompressible, stone-filled gizzard.

We are not able to provide a plausible explanation for the high prevalence of transverse and oblique fractures. Assuming that butterfly and comminuted fractures are mostly caused by an external impact, at least some of the transverse and oblique fractures might occur spontaneously, e.g., pathological fractures, a possibility raised previously (1). Pathological fractures might be expected with relatively strong muscles contracting against weak bone and are supported by the fact that these fractures frequently resembled fractures reported in mammals with primary or secondary hyperparathyroidism (28). It may be assumed that any bone damage is a combination of decreased bone strength and internally and externally applied forces, a possibility supported by observations of increased fracture susceptibility with decreased keel bone mineral density (29).

### Fracture Development

Most keel bone fractures in this study occurred when hens were 31–33 weeks of age, a period just after peak of lay where hens have been laying eggs regularly for approximately 10 weeks. When reaching sexual maturity at 16–18 weeks of lay, a hen's bone metabolism undergoes dramatic changes: production of structural bone ceases (30), the elasticity of the bones decreases (31), and calcium retention efficiency increases (32). The loss of bone elasticity might be insufficiently compensated by the increase in calcium retention around the age of 31–33 weeks, leading to an increase in the incidence of fractures. The subsequent decrease in new fractures during the following weeks could partly be explained by the increase of calcium retention, which would solidify the bone. Alternatively, the high incidence of new fractures at 31–33 weeks of age results in callus formation, which deforms the bone and makes it thicker. This would not only explain the lower incidence of KBF for hens older than 33 weeks but would also explain the higher incidence of butterfly and comminuted fractures, as more severe trauma would be needed to cause these types of fracture.

Another hypothesis to explain the higher incidence of KBF at the age of 31–33 weeks would be that, after moving the hens from the rearing to the laying barn, hens need to adapt to their new environment and might use the lower perches of the aviary less than the upper perches. Indeed, use of the upper perches increases with increasing age (13) and was found to be a risk factor for KBF (20). After a linear increase in KBF incidence leading to a peak at P4, this will reduce again due to a lower use of the perches. Indeed, the prevalence of KBF was then found to be high, and hens with keel bone lesions showed reduced mobility in moving between tiers and associated perches (13). Further research is needed to confirm these hypotheses, though we believe our methodology contributes to this effort.

Fractures located in the region of the breast muscles (A and B, used during flight) as well as fractures in the region of the keel bone exposed while approaching the perch (C and E) would be expected to cause more pain than fractures in region D, as more forces are applied. Comminuted fractures and fractures with dislocated fragments leading to a long healing period were also suspected to be of greater importance regarding the welfare of the hen when compared to smaller and non-dislocated fractures at the ventral aspect of the keel bone. These expectations were not evaluated in our study, though we believe the development of the described methodology is a critical first step toward those goals. Our effort was also unique in allowing for longitudinal observations of damage in a comparable manner. Radiography allowed multiple age- and hen-specific images to be overlaid and features of interest to be compared. By making these comparisons within hens over time, our efforts found the duration of healing ranged from 0 (radiographically already healed at detection) to 36 weeks with 85% of fractures healing within 7 weeks, results that are in accordance with Richards et al. (9). Fractures that did not heal or required extended time to heal, often lasting several months without evidence of healing, are also a known concern in mammals. Explanations for delayed or absent fracture healing are missing healing stimuli either due to a lack or cloying (micro-) motion at the fracture ends or a too large fracture gap due to extensive fragment dislocation (33). Additionally, decreased primary osteoclastic and osteoblastic activity might influence fracture healing. All these conditions can lead to atrophic or hypertrophic non-union of fragments. The concept of fracture treatment involves fragment repositioning, stabilization/fixation, and restriction of motion, measures which have not been an option in our quasi-commercial study setting or that of a standard production environment. Spontaneous fracture healing in wild animals is limited by persisting motion and limping. In analogy to fracture healing in wild animals, spontaneous fracture healing in our focus animals mostly lead to mal-union and shortening of the keel bone (34).

### Non-fracture Lesions

Fifty-five percent of the non-fracture lesions and 45% of the sclerosis developed in a later observation period to a fracture. Other non-fracture lesions were angulations, predominantly in section E, and indentations, predominantly in section AB. The occurrence and development of such lesions might support the hypothesis of the presence of decreased bone strength and therefore high susceptibility to any kind of damage. In this context, indentations might be the result of chronic external pressure on the keel applied when the hens are sitting on the perch (2).

### Study Limitation

In the current study, only a laterolateral radiograph of the keel bone was performed, which is a major shortcoming as an accurate image interpretation should involve at least two projections at 90° to each other. The authors are aware that a single projection would lead to some lesions (e.g., deviations) being missed, underestimated (direction and amount of dislocation and angulation) or misinterpreted [fracture gap, callus formation, (35)]. Even though two projections at an angle of 90° to each other is necessary, preliminary efforts in a pilot study demonstrated that a craniocaudal tangential or ventrodorsal projection proved ineffective due to superimposition.

## Conclusions

The described radiographic protocol for keel bone lesions is suitable for longitudinal on-site examinations. Keel bone fractures appear more frequent than reported in earlier studies which we believe relates to our protocol's superior ability to assess damage. Further investigations should be conducted to understand the clinical significance (e.g., activity, productivity, pain) as well as the cause for damage using the described technique generating detailed representations of keel damage.

## Data Availability Statement

All datasets generated for this study are included in the article/Supplementary Material.

## Ethics Statement

The use of radiography for animal experimentation was reviewed and approved by the Swiss radiation authority (Approval number BE-03222.41.013). Approval for use of experimental animals was obtained from the Veterinary Office of the Canton of Bern in Switzerland (approval number BE31/15). The experiment complied with Swiss regulations regarding the treatment of experimental animals.

## Author Contributions

SB produced the radiographs, analyzed data, was the principal developer of the scoring-system, and was the principal author of the manuscript. CR was the principal organizer of the daily operations for the parallel study as part of a doctoral research program, provided important information on study design, and performed the statistics used for the tables and Figure 9. MT was principal supervisor of the parallel project and was also the recipient for the associated funding. UG supervised this project, gave major inputs regarding radiographic evaluation and development of the scoring system, and was responsible for radiographic quality and radiation protection. All authors reviewed the manuscript and approved the submitted version.

### Conflict of Interest

The authors declare that the research was conducted in the absence of any commercial or financial relationships that could be construed as a potential conflict of interest.

## Supplementary Material

Data Sheet S1 Raw data used for the study.

## Abbreviations

LB: Lohmann Brown
LSL: Lohmann Selected Leghorns
KBF: Keel bone fracture
P: Observation Phase
SD: Standard deviation.
